# To the beat of a different drum: determinants implicated in the asymmetric sequence divergence of *Caenorhabditis elegans* paralogs

**DOI:** 10.1186/1471-2148-13-73

**Published:** 2013-03-27

**Authors:** Vaishali Katju

**Affiliations:** 1 Department of Biology, University of New Mexico, Albuquerque NM 87131, USA

## Abstract

**Background:**

Gene duplicates often exhibit asymmetric rates of molecular evolution in their early evolutionary existence. This asymmetry in rates is thought to signify the maintenance of the ancestral function by one copy and the removal of functional constraint on the other copy, enabling it to embark on a novel evolutionary trajectory. Here I focused on a large population of evolutionarily young gene duplicates (K_*S*_ ≤ 0.14) in the *Caenorhabditis elegans* genome in order to conduct the first combined analysis of four predictors (evolutionary age, chromosomal location, structural resemblance between duplicates, and duplication span) which may be implicated in the asymmetric sequence divergence of paralogs at the nucleotide and amino acid level. In addition, I investigate if either paralog is equally likely to embark on a trajectory of accelerated sequence evolution or whether the derived paralog is more likely to exhibit faster sequence evolution.

**Results:**

Three predictors (evolutionary age of duplicates, chromosomal location and duplication span) serve as major determinants of sequence asymmetry between *C. elegans* paralogs. Paralogs diverge asymmetrically in sequence with increasing evolutionary age, the relocation of one copy to a different chromosome and attenuated duplication spans that likely fail to capture the entire ancestral repertoire of coding sequence and regulatory elements. Furthermore, for paralogs residing on the same chromosome, opposite transcriptional orientation and increased genomic distance do not increase sequence asymmetry between paralogs. For a subset of duplicate pairs wherein the ancestral versus derived paralog could be distinguished, the derived paralogs are more likely to evolve at accelerated rates.

**Conclusions:**

This genome-wide study of evolutionarily young duplicates stemming primarily from DNA-mediated small-scale duplication events demonstrates that genomic relocation to a new chromosome has important consequences for asymmetric divergence of paralogs, akin to paralogs arising from RNA-mediated duplication events. Additionally, the duplication span is negatively correlated with sequence rate asymmetry among paralogs, suggesting that attenuated duplication spans stemming from incomplete duplication of the ORF and/or ancestral regulatory elements further accelerate sequence divergence between paralogs. Cumulatively, derived copies exhibit accelerated rates of sequence evolution suggesting that they are primed for a divergent evolutionary trajectory by changes in structure and genomic context at inception.

## Background

Gene duplication has long been thought to generate the genetic fodder for the appearance of novel biochemical traits ultimately linked to the origin of phenotypic diversity. Analyses of whole-genome sequence data from newly published genomes since the late 1990s has confirmed the ubiquitous presence of gene duplicates in diverse taxa (reviewed in [1]). Hand-in-hand has come the emerging recognition that genic and genomic duplications lead to an extensive amplification of genome size and the creation of gene-families with multiple members [2,3]. The rapid advancement in molecular techniques that enable genome-wide analyses of DNA content has uncovered immense copy-number variation (CNV henceforth) in multiple genomes [4-11]. Recent whole-genome sequencing of *Saccharomyces cerevisiae*[12] and comparative genome hybridization of *Caenorhabditis elegans*[13] experimental lines maintained under strict bottlenecking conditions to permit the accumulation of mutations under relaxed selective constraints have provided the most robust direct estimates of the spontaneous gene duplication rate. Despite the technical differences in the methods used to detect CNV in these aforementioned studies, both provide strong evidence for astoundingly high genome-wide rates of spontaneous gene duplication that exceed the base substitution rate by one hundred to ten-thousand fold. Indeed, the contribution of CNVs and other structural variants to intraspecific polymorphism is now thought to vastly exceed that of SNPs [5].

Despite these advances, the processes contributing to the preservation of a new gene duplicate under a barrage of potentially deleterious mutations and its trajectory towards the acquisition of a new function or pseudogenization remain obscure. At the simplest level, widely-cited models of gene duplicate evolution predict that the altered functional fates of paralogs are expected to manifest as asymmetric rates of molecular evolution given the expected correlation between sequence and functional divergence [14,15], a correlation that has been borne out by recent studies [16-23]. Ohno’s model of gene duplication additionally predicted that the two paralogs are expected to have asymmetric rates of molecular evolution, with the ancestral copy evolving under purifying selection and the redundant copy experiencing accelerated rates of evolution due to a regime of relaxed selective constraints and/or positive selection for a new function [14,24]. Multiple researchers turned their attention to measuring the degree of sequence divergence among paralogs in a diverse set of genomes to determine whether paralogs, on average, exhibit symmetrical or asymmetrical rates of molecular evolution. The earliest studies found paralogs to exhibit uniform/symmetrical rates of molecular evolution [25-27], which was initially taken as evidence against Ohno’s model of gene duplicate evolution. However, these initial studies did focus on evolutionarily older gene paralogs such as those in *Arabidopsis* with a putative origin dating back to 100 MYA [27] wherein the molecular signature of rate asymmetry early in their evolutionary existence could have been masked by the subsequent accumulation of additional mutations with increasing evolutionary age [24]. Subsequent studies focusing on evolutionarily younger gene duplicates have demonstrated an appreciable frequency of rate asymmetry among paralogs [28-32]. Rate asymmetry between paralogs has also been demonstrated for more ancient duplicates in eight yeast species following a whole-genome duplication event in their common ancestor [33].

The majority of studies investigating the rates of molecular divergence of paralogous genes have reported evidence for the frequent incidence of asymmetric sequence divergence between the gene copies. However, we still have little insight into what genomic factors, if any, facilitate this divergence. A handful of studies have further attempted to investigate what factors may promote asymmetric sequence divergence of paralogs. A study of rodent (rat and mouse) paralogs was the first to systematically investigate paralog rate asymmetry as a function of the mechanism of duplication and the degree of genomic proximity [30] and demonstrated that (i) retrogenes undergo accelerated evolution relative to their static paralogs, and (ii) genomic relocation of one copy, irrespective of the mechanism of duplication, precipitates greater sequence rate asymmetry between paralogs. A similar study in five mammalian genomes (chimp, dog, human, mouse and rat) found that paralogs stemming from retrotransposition and distant DNA-mediated duplication events are more likely to evolve asymmetrically relative to tandemly located ones [31]. Furthermore, duplicated genes that had relocated to new chromosomal locations in the human, macaque, mouse and rat genomes were more likely to undergo positive selection than the static copy [34].

All of these studies have focused on mammalian genomes that tend to have retrotransposition as one of the dominant mechanisms for the origin of duplicate genes. As such, these studies have emphasized the role of DNA-based versus RNA-based mechanisms of gene duplication in the asymmetric sequence divergence of paralogs. It is yet undetermined if a similar or divergent set of characters influence paralog rate asymmetry in genomes where DNA-mediated duplication events dominate the duplication landscape. Furthermore, additional genomic and structural characteristics of paralogs such as transcriptional orientation, duplication span, and the degree of structural resemblance between paralogs may influence sequence asymmetry of paralogs, but to date remain unexplored. This study has two major goals, namely (i) to quantify the degree of asymmetry in molecular rates for putative evolutionarily young gene duplicates in the *Caenorhabditis elegans* genome which stem primarily from DNA-based duplication events [35,36], and (ii) to assess the dependence of sequence rate symmetry/asymmetry of *C. elegans* paralogs on a suite of evolutionary or genomic characteristics to determine which particular features predispose them to divergent evolutionary trajectories in their early evolutionary existence with potential consequences for functional divergence.

## Methods

### Identification of *C. elegans* paralogs, accession of sequences and other genic information

Lynch and Conery [2] had initially identified paralogs in the *C. elegans* genome [37] by downloading the complete set of available putative amino acid sequences, filtering out all possible nonfunctional protein sequences and conducting all-against-all BLASTP searches with E = 10^-10^ as a cutoff to identify duplicate pairs. To protect against the inclusion of large multigene families, only small gene families with ≤ 5 duplicate pairs were retained. From this dataset, 290 gene duplicate pairs with low synonymous divergence were further analyzed to determine their genomic and structural characteristics [35].

Of the initial 290 gene duplicate pairs with low synonymous site divergence (*K*_*S*_ ≤ 0.10) within the *C. elegans* genomic data set of Katju and Lynch [35,36], 63 of the initial 290 pairs were no longer consider valid paralogs in Wormbase WS214 owing to one of the following conditions: (i) alterations to the ORF(s) of one or both paralogs such that they no longer appeared homologous in their coding regions, or (ii) one or both paralogs retired/killed/superseded (e.g. following identification as a transposon). These 63 duplicate pairs were removed from the dataset. Spliced, unspliced and 2 kb of the flanking region (both 5^′^ and 3^′^) nucleotide and amino acid sequences for both paralogs for the remainder 227 duplicate pairs were retrieved from WormBase release WS214 (http://www.wormbase.org/). Paralogous sequences were aligned using ClustalW2 at the EMBL-EBI site and checked manually in the sequence alignment editor Se-Al [http://tree.bio.ed.ac.uk/software/seal/]. With respect to the nucleotide sequence alignments, 2 kb of upstream and downstream flanking region sequence in addition to the ORF sequence were initially retrieved and aligned with the spliced and unspliced sequences. For instances wherein homology between paralogs extended beyond 2 kb of the flanking region(s), an additional 1 kb of flanking sequences was accessed from the database and subsequently aligned. The addition and alignment of flanking sequences was iterated until no homology was apparent between the paralogs for a continuous stretch of 1 kb in both the 5^′^ and 3^′^ directions.

### Quantifying the molecular sequence divergence of paralogs

Measures of synonymous sequence divergence in coding regions (*K*_*S*_) were recalculated using the codeml program in the PAML package [38] via PAL2NAL (http://www.bork.embl.de/pal2nal/). For each duplicate pair, I attempted to identify an outgroup gene that exists as a single-copy ortholog in a closely-related genome (*C. brenneri*, *C. briggsae*, *C. japonica* or *C. remanei*) or a more evolutionarily distant paralog in the same multigene family within *C. elegans*. 97 duplicate pairs lacking an identifiable ortholog in the four congeneric outgroup genomes or a more distantly-related gene family member in *C. elegans* were excluded from further analysis. An outgroup sequence was successfully identified for the remaining 130 of the 227 duplicate pairs which comprised the final data set. The synonymous divergence between paralogs within this set of 130 duplicate pairs ranged from 0 – 13.6% (0≤ *K*_*S*_ ≤ 0.1363).

Tajima [39] proposed a relative rate test to determine whether two protein or nucleotide sequences have evolved at a similar relative rate. These two sequences could be orthologs from two organisms or paralogs within the same organism. In other words, the relative rate test statistically determines if two sequences follow the molecular clock hypothesis of approximately constant rates of nucleotide or amino acid substitution over evolutionary time. In the relative rate test, sequences A and B share a common ancestor O and the sequence of an outgroup (C) is known. By measuring the substitution rates AB, AC, and BC, it is possible to infer the rates OA and OB and to perform a *χ*^*2*^-test to determine whether these rates are comparable (the null hypothesis) or whether one lineage has evolved at a relative accelerated or decelerated rate, thus violating the behavior of a molecular clock. Within the final set of 130 *C. elegans* duplicate pairs, each sequence triplet comprising the homologous coding sequences of two focal *C. elegans* paralogs and an outgroup sequence was aligned at the protein and nucleotide levels and analyzed via the relative rate test [39] using the program MEGA 4.0 [40] (http://www.megasoftware.net/). For gene duplicate pairs displaying structural heterogeneity in their coding regions (*partial* and *chimeric* structure discussed in the subsequent section), all measures of sequence divergence (synonymous divergence K_*S*_ and degree of sequence asymmetry/symmetry via Tajima’s relative rate test) were calculated using only the homologous regions between the focal *C. elegans* paralogs.

Conservative tests like Tajima’s relative rate test have extremely low statistical power for detecting rate asymmetry between paralogs that have accumulated few mutations, as would be the case for evolutionarily recent duplicates [24]. For example, Lynch and Katju [24] calculated that if each of two paralogs had accrued ten mutations since the duplication event, an absolute difference of at least nine mutations between the two copies would be required to reject the null hypothesis of equal rates. The power to detect asymmetric sequence divergence is also compromised in shorter tracts of paralogous sequences [41]. Furthermore, the earliest-incurred mutations may be paramount in dictating altered evolutionary trajectories for individual paralogs. To circumvent this challenge of low power associated with Tajima’s relative rate test, a new continuous variable was created to measure the extent of asymmetry between duplicates using the number of unique sites in each paralog as determined by Tajima’s relative rate test. Tajima’s relative rate test was employed to determine the number of unique sites in each paralog relative to an outgroup sequence. But in lieu of restricting our final data set to an extremely low number of duplicate pairs that were detected by Tajima’s relative rate test as showing significant rate asymmetry, all 130 duplicate pairs were used in the analyses. This variable, “*asymmetry/site”* was quantified at the level of both the nucleotide and amino acid sequences and was calculated as the absolute difference in the number of unique sites for each paralog as determined by Tajima’s relative rate test and subsequently scaled to a per site level to standardize for gene length. An *asymmetry/site* value of 0 indicates equal rates of molecular evolution in the paralogs. This method also serves to effectively exclude nonhomologous sites present in one paralog to the exclusion of the other (such as those found in *partial* and *chimeric* duplicate pairs). These measures of asymmetry were calculated for each of 130 duplicate pairs (Additional file 1: Table S1 and Additional file 2: Table S2) and lent far greater power to the study, enabling a more comprehensive analysis of the determinants of rate asymmetry than would have been possible if only duplicate pairs demonstrating significant rate asymmetry based on Tajima’s relative rate test were utilized.

### Genomic features of paralogs

Data on the following structural and genomic features of the *C. elegans* paralogs was collected, namely (i) the structural resemblance between paralogs, (ii) genomic proximity (same *vs.* different chromosome(s)), (iii) duplication span under the assumption that lengthier duplication tracts likely capture the entire ancestral coding region and regulatory motifs, and (iv) the K_*S*_ (the number of synonymous substitutions per synonymous site). Although data on the genomic attributes of focal paralogs were collected in a previous study [35], genomic sequence information deposited on online bioinformatic databases as WormBase are subject to frequent reannotation. Hence, the entire analysis was repeated using sequence and genomic information retrieved from WormBase release WS214. The alignment and comparison of paralog sequences as detailed in Katju and Lynch [35] enabled the identification of duplication termination points and the degree of structural resemblance between paralogous sequences. Briefly, *complete* duplicates share sequence homology throughout their open reading frame, barring small indels, if present. *Partial* and *chimeric* duplicates comprise pairs wherein one or both paralog(s) have unique exon(s) to the exclusion of the other copy, respectively. In this study, duplicates were classified as having one of two categories of structural resemblance, namely (a) homogeneous duplicates (*complete*), or (b) heterogeneous duplicates (*partial* or *chimeric*). With respect to genomic location, paralogs were initially classified as residing on (a) the same chromosome, or (b) different chromosomes. For the subset of gene duplicate pairs with both paralogs residing on the same chromosome, two additional measures of genomic location were assessed, namely (a) the genomic distance (in bp) between the two paralogs, and (b) the transcriptional orientation of each paralog. Duplication span was calculated as the length of sequence homology (in bp) shared between the two paralogs between their duplication termination points. In the event that a paralog accumulated one or several indels within its region of homology with the other paralog, two values of duplication span were calculated by treating each paralog as the ancestral copy. The lower, conservative value of these two duplication spans was included in the analysis.

### *Assessing the dependence of rate asymmetry on the genomic and structural features of paralogs*

This study utilized a multiple regression approach to determine the best set of predictor variables that explain rate asymmetry among paralogs at the sequence level. The standardized variable *asymmetry/site* was considered as the *y* variable. The four predictor variables labeled *x*_*1*_ through *x*_*4*_ are as follows: (a) *x*_*1*_ = category of structural resemblance between paralogs; nominal variable with two categories termed “*homogeneous*” (*complete* duplicates) versus “*heterogeneous*” (*partial* and *chimeric* duplicates), (b) *x*_*2*_ = chromosomal location of the two paralogs; nominal variable with two categories “*same*” versus “*different*” for same chromosome and different chromosomes, respectively, (c) *x*_*3*_ = duplication span; numeric, continuous variable, and (d) *x*_*4*_ = K_*S*_ ; fraction of synonymous substitutions per synonymous site; numeric, continuous variable.

The multiple regression approach aims to best predict the value of the *y* variable with the smallest subset of *x* predictor variables. While increasing the number of predictor variables result in a greater ability to explain the variance in *y*, it can lead to a decline in the predictive ability of the model and greater multicollinearity between predictor variables. In order to exclude predictor variables with limited contribution towards explaining the variance in *y*, a stepwise linear multiple regression procedure using mixed selection was utilized. At each step, the variable with the highest significance was added to the regression model. The process selected variables that were significant at the 0.15 level for entry and a 0.10 probability to leave. The process was terminated when no additional variables were significant. Once the stepwise selection process completed selecting the variables, the model was run using a standard least squares regression with the effects that were selected by the stepwise process as most active. All regression analysis was conducted using JMP® software, v. 5.0.1.2.

### Do derived copies evolve faster than their ancestral counterparts?

Ohno originally hypothesized that gene duplication enables redundancy, thereby allowing one gene copy to explore new evolutionary space by accumulating mutations [14]. Because newborn gene duplicates were thought to be redundant to the ancestral copy with respect to sequence and functionality, Ohno’s model also implicitly assumes that the either copy had the evolutionary potential to maintain the ancestral role or embark on a novel trajectory. Here the null hypothesis is one of no difference in the frequencies of ancestral versus derived copies bearing signatures of faster sequence evolution. ‘Derived’ and ‘ancestral’ in the context of this study refer to the location of paralogs in the genome rather than function. For a subset of 37 pairs within the original dataset, I was able to assign ancestral versus derived copy status to the two paralogs based on structural and/on syntenic comparisons to a single-copy ortholog in *C. briggsae*. In a previous study, I had compared the exon-intron structure of structurally heterogeneous *C. elegans* paralogs (*partial* or *chimeric*) to that of a single-copy ortholog in *C. briggsae,* if present [36]. The *C. elegans* paralog bearing greater structural similarity to the *C. briggsae* ortholog was taken to represent the ancestral locus. Additionally, I also identified all *C. elegans* duplicate pairs in this study that (i) comprised paralogs residing on different chromosomes, and (ii) had available a single-copy ortholog in *C. briggsae* as an outgroup. The genomes of *C. brenneri*, *C. japonica* and *C. remanei* have not been fully annotated and only bear the contig-specific information for the focal orthologs. While chromosomal translocations can and do alter the ancestral genomic position of gene duplicates, this study parsimoniously assumed that shared synteny (chromosomal location) between a *C. elegans* paralog and the *C. briggsae* ortholog represents the ancestral genomic location of the progenitor copy in the *C. elegans* genome. Hence, the *C. elegans* paralog located on the same chromosome as the *C. briggsae* ortholog was taken to represent the ancestral copy. The *standardized asymmetry* (at both the nucleotide and amino acid level) was taken to equal [(number of unique sites in the ancestral copy – number of unique sites in the derived copy)/ length of homologous coding sequence between the two paralogs). The distribution of this difference was tested with a Wilcoxon signed-ranks test to determine if, on average, paralogs in ancestral and derived locations in the genome are equally likely to exhibit accelerated sequence evolution.

## Results

### Detection of significant sequence asymmetry by Tajima’s relative rate test increases with evolutionary age of gene duplicates

Of the 130 *C. elegans* gene duplicates pairs resulting from small-scale duplications (synonymous divergence values ranging from 0 to 13.6%), 17 (~13.1%) displayed significant sequence asymmetry at the nucleotide level based on Tajima’s relative rate test (significance level of α = 0.05). At the amino acid level, significant sequence asymmetry was detectable in only 9 of 130 (~7%) duplicate pairs. 7 of these 9 pairs (~78%) with significant amino acid sequence asymmetry also displayed asymmetry at the nucleotide level.

**Figure 1:**
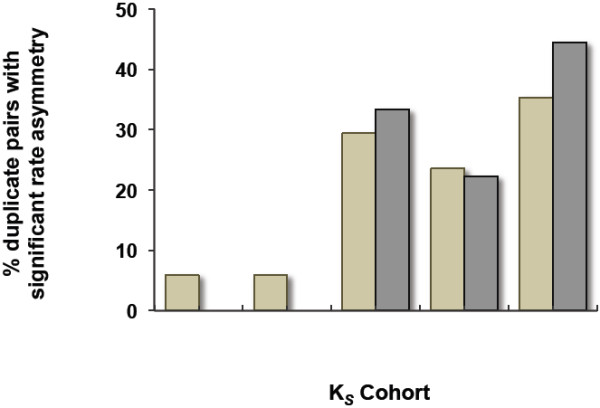
**Percent *C. elegans *gene duplicate pairs displaying significant nucleotide (beige) and amino acid (grey) rate asymmetry by Tajima’s relative rate test within each of five K**_***S ***_**cohorts.** Age cohorts 0 ≤ K_*S*_ < 0.01, 0.01 ≤ K_*S*_ < 0.03, 0.03 ≤ K_*S*_ < 0.05, 0.05 ≤ K_*S*_ < 0.08, and 0.08 ≤ K_*S*_ < 0.14 are represented by 24, 29, 29, 32, and 16 duplicate pairs, respectively.

At the nucleotide level, 16 of the 17 (~94%) gene duplicates showing significant sequence asymmetry using Tajima’s relative rate test had K_*S*_ values exceeding 0.0299. Figure 1 displays the percent of gene duplicates pairs with significant sequence asymmetry at the nucleotide level within five age-cohorts classified according to their degree of synonymous divergence per synonymous site (0.00 ≤ K_*S*_ < 0.01, 0.01 ≤ K_*S*_ < 0.03, 0.03 ≤ K_*S*_ < 0.05, 0.05 ≤ K_*S*_ < 0.08, and 0.08 ≤ K_*S*_ < 0.14). These results suggest that sequence asymmetry at the DNA sequence level is positively correlated with increasing evolutionary age. Indeed, there exists a significant positive correlation between K_*S*_ and standardized asymmetry at the DNA level across the complete dataset of 130 gene duplicate pairs used in this study (*n* = 130 duplicate pairs; *Kendall’s tau* = 0.244; *p* < 0.0001). Likewise, standardized asymmetry at the amino acid level is positively correlated with increasing evolutionary age of gene duplicates (Figure 1) (*n* = 130 duplicate pairs; *Kendall’s tau* = 0.163; *p* < 0.0074).

The above results are not unexpected. Indeed, the magnitude of sequence asymmetry between paralogs is expected to increase with evolutionary time due to a gradual linear accumulation of mutations and the stochastic nature of mutations. These results are presented as formal evidence that the potential for sequence asymmetry between paralogs is positively correlated with increasing evolutionary age. Furthermore, the fact that Tajima’s relative rate tests only detect significant sequence asymmetry between paralogs with K_*S*_ ≥ 0.03 serves to highlight the limited power of the test in detecting significant asymmetry in paralogs with extremely recent evolutionary origins.

### Pseudogene containing duplicate pairs do not exhibit greater asymmetry at the sequence level

**Figure 2:**
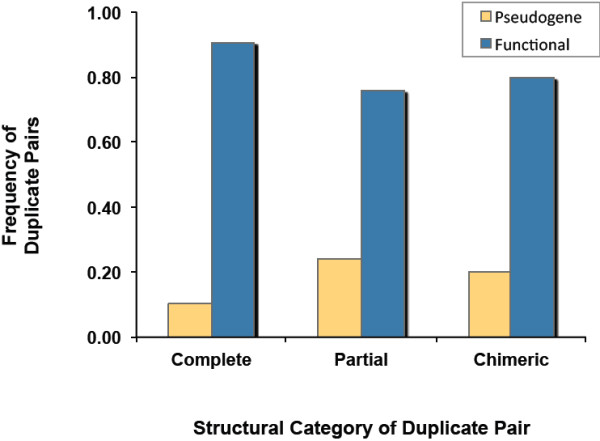
**Composition frequencies of pseudogenized versus functional gene duplicate pairs within three structural categories of gene duplicates (*complete *duplicates, *n *= 65; *partial *duplicates, *n *= 25; *chimeric *duplicates, *n *= 40).** Pseudogenized pairs comprised cases wherein one or both paralogs are classified as pseudogenes on WormBase; functional pairs comprise instances where both paralogs have a putative or known function.

20 of 130 gene duplicate pairs were characterized by WormBase as possessing one or both paralog(s) designated as pseudogenes. Although gene duplicates with *partial* or *chimeric* structural resemblance were two-fold more likely to have a paralog annotated as a pseudogene relative to *complete* duplicate pairs (Figure 2), a *G*-test for goodness of fit revealed no significant difference in the frequencies of pseudogenes among the three structural classes of gene duplicates (*complete*, *partial* and *chimeric*) (*G*_*adj*_ = 3.86; *df* = 2, 0.1 <*p* < 0.5).

**Figure 3:**
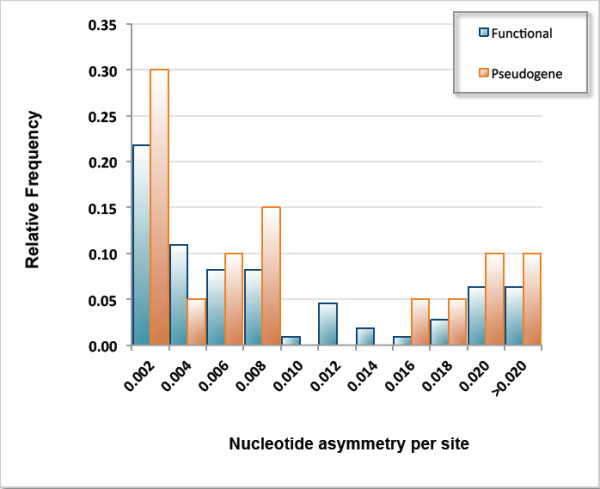
**Relative frequency distributions of nucleotide asymmetry per site for gene duplicate pairs designated functional (*n *= 110 pairs) or as pseudogene(s) (*n *= 20 pairs).**

Theory predicts that pseudogenes, because they lack function, are freed from selective constraints and hence accumulate mutations at an accelerated pace that mirrors the rate of spontaneous mutations [42,43]. If pseudogenes do indeed have accelerated rates of sequence evolution, one might expect greater asymmetry among paralogs at the sequence level for duplicate pairs comprising pseudogene(s) relative to their counterparts possessing two putatively functional paralogs. However, Wilcoxon two-sample tests detected no significant difference in sequence asymmetry between duplicate pairs comprising pseudogene(s) relative to pairs with both putatively functional paralogs at both the nucleotide level (*Wilcoxon two-sample test*: *Z* = 1.583; *p* = 0.1135) (Figure 3) and the amino acid level (*Wilcoxon two-sample test*: *Z* = 1.111; *p* = 0.2667). Furthermore, the gene duplicate pairs containing pseudogenes within this dataset are not disproportionately younger than their functional counterparts. However, the number of pseudogenes within this dataset is small and failure to detect rate asymmetry amongst them may be a consequence of low statistical power.

### Duplication span, chromosomal location and K_S_ significantly influence rate asymmetry between paralogs at the nucleotide level

A forward stepwise multiple regression model was used to examine the influence of four potential explanatory variables (predictors) on asymmetry in nucleotide sequence divergence (as measured by *nucleotide asymmetry/site*) of paralogs for 130 duplicate pairs. Three of the four predictors constitute key genomic and structural characteristics of the paralogs in question, namely (i) structural resemblance between paralogs (homogeneous = *complete* duplicates; heterogeneous duplication = *partial* or *chimeric* duplicates), (ii) chromosomal location (same *versus* different chromosome(s)), and (iii) duplication span in bp. Because preceding analysis demonstrated that sequence rate asymmetry tends to be positively correlated with the evolutionary age of gene duplicates as determined by the degree of synonymous divergence per synonymous site, I further included K_*S*_ as the fourth predictor in the regression model.

The Summary of Fit section in Table 1 shows that the model accounted for 19.43% of the variation around the mean (R-square). The remaining residual error was estimated to have a standard deviation of 0.0098 (root mean square error). Additionally, a full model fit was compared to the simple mean model fit. The Analysis of Variance section in Table 1 lists the sums of squares and degrees of freedom used to form the whole model test utilizing a *F*-test; the Error, C. Total and Model sum of squares are the ingredients required to test the whole-model hypothesis that all the parameters in the model are zero except for the intercept (the simple mean model). The *F*-ratio of 10.13 is highly significant (*p* < 0.0001) which indicates that the whole model comprising three predictors/parameters does provide a significantly better fit to the data than simply the mean.

**Table 1:** **Results from a stepwise multiple linear regression model evaluating four regressor/explanatory variables as predictors of asymmetric rates of nucleotide sequence evolution between paralogs of 130 *C. elegans *gene duplicate pairs**

**Summary of fit:**					
R-square				0.1943	
R-square adjusted				0.1751	
Root mean square error				0.0098	
Mean of response				0.0069	
Observations				130	
Analysis of variance:					
*Source*	*df*	*Sum of squares*	*Mean square*	*F-ratio*	*p-value*
Model	3	0.00294	0.000981	10.1283	< 0.0001
Error	126	0.01220	0.000097		
C. Total	129	0.01514			
Parameter estimates:					
*Term*	*Estimate*	*Std. Error*	*t ratio*	*p-value*	
Intercept	0.005447	0.001834	2.97	0.0036	
Chromosomal location (different)	0.002620	0.000997	2.63	0.0097	
Duplication span	−0.000001	4.5e-7	−2.55	0.0120	
K_*S*_	0.121371	0.026453	4.59	<0.0001	

**Figure 4:**
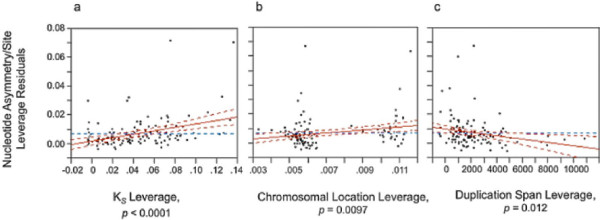
**Partial regression leverage plots from a stepwise regression analysis detailing the contribution of three significant predictors to standardized nucleotide asymmetry between paralogs comprising 130 *C. elegans *duplicate pairs.** The vertical axis represents the partial residuals of standardized nucleotide asymmetry (*y* variable) and the horizontal axis represents the partial residual of the specific predictor variable. Dashed red lines represent 95% confidence curves for the full model (solid red line) based on actual data. The dashed blue horizontal line indicates a model with no effect. Confidence curves enclosing a solid red line deviating significantly from the dashed blue horizontal line indicate a significant effect of the predictor variable. Alternatively, when the confidence curves enclose the dashed blue horizontal line, the effect of the predictor variable is nonsignificant. (**a**) Partial regression leverage plot showing a significant positive effect of evolutionary age of duplicates (K_*S*_) on nucleotide rate asymmetry between paralogs. (**b**) Partial regression leverage plot showing a significant positive effect of chromosomal location on nucleotide rate asymmetry between paralogs. Rate asymmetry between paralogs increases with relocation of one copy to a different chromosome. (**c**) Partial regression leverage plot showing a significant negative effect of duplication span on nucleotide rate asymmetry between paralogs.

The Parameter Estimates section in Table 1 demonstrates three predictors to be highly significant with respect to their influence on nucleotide sequence asymmetry/site among the *C. elegans* paralogs, namely (i) the chromosomal location of paralogs, (ii) duplication span and (iii) the evolutionary age of paralogs (K_*S*_). The structural degree of resemblance between paralogs (*complete*, *partial* or *chimeric*) did not display a significant effect on rate asymmetry between paralogs, at least over their homologous regions. Sequence asymmetry between paralogs at the nucleotide levels increases with (i) evolutionary age of the paralogs, (ii) the relocation of one paralog to another chromosome, and (iii) an attenuated duplication span (Figure 4).

All four predictor variables were further investigated to test for any association between them (six pair-wise comparisons). The tests employed are different based on whether both variables were continuous, categorical or one continuous and one categorical. There was no association between *K*_*S*_ and (i) duplication span (*Kendall’s τ* = − 0.0091, *p* = 0.8784), (ii) structural resemblance between paralogs (*t* = −1.832, *p* = 0.0692), or (iii) chromosomal location of paralogs (*t* = −1.417, *p* = 0.1588). Likewise, there was no association between structural resemblance of paralogs and (i) their chromosomal location (χ^2^ = 0.087, *p* = 0.7682), or (ii) the duplication span (*t* = − 1.392, *p* = 0.1662). The only predictor variables with a significant association between them are duplication span and chromosomal location (*t* = 2.718, *p* = 0.0075), where duplication spans of paralogs residing on different chromosomes is greater than that of paralogs residing on the same chromosomes (mean duplication spans of 2979 and 1940 bp, respectively). However, asymmetry between paralogs (at both the nt and aa levels) decreases with increased duplication span but increases with distribution of paralogs to different chromosomes. Hence the relationship between duplication span and chromosomal location cannot account for the two significant associations in the multiple regression analyses between asymmetry and duplication span on one hand, and asymmetry and different chromosomal location on the other. In other words, even though duplications involving different chromosomal location of paralogs tend to have larger duplication spans, paralogs still exhibit greater asymmetry when located on different chromosomes.

### Duplication span, chromosomal location and K_S_ similarly influence rate asymmetry between paralogs at the amino acid level

Similarly, a forward stepwise multiple regression model was used to examine the influence of the four previously described predictors on asymmetry in amino acid sequence divergence (as measured by *amino acid asymmetry/site*) of paralogs for 130 duplicate pairs. The results for sequence asymmetry at the amino acid level (Table 2) are in concordance with those of nucleotide sequence asymmetry, with the same three predictors (chromosomal location, duplication span and K_*S*_) showing significant effects on rate asymmetry at the amino acid level. The structural degree of resemblance between paralogs had no effect on paralog rate asymmetry at the amino acid level.

**Table 2:** **Results from a stepwise multiple linear regression model evaluating four regressor/explanatory variables as predictors of asymmetric rates of amino acid sequence evolution between paralogs of 130 *C. elegans *gene duplicate pairs**

**Summary of fit:**					
R-square			0.1322		
R-square adjusted			0.1115		
Root mean square error			0.0228		
Mean of response			0.0136		
Observations			130		
Analysis of variance:					
*Source*	*df*	*Sum of squares*	*Mean square*	*F-ratio*	*p-value*
Model	3	0.01002	0.003341	6.3983	0.0005
Error	126	0.06580	0.000552		
C. total	129	0.07582			
Parameter estimates:					
*Term*		*Estimate*	*Std. error*	*t ratio*	*p-value*
Intercept		0.013279	0.004258	3.12	0.0023
Chromosomal location (different)		0.005298	0.002316	2.29	0.0238
Duplication span		−0.000003	0.000001	−2.53	0.0127
K_*S*_		0.200085	0.061427	3.26	0.0014

### Transcriptional orientation and genomic distance on the same chromosome have no discernible influence on sequence asymmetry among paralogs

It is possible that a greater genomic distance between paralogs on the same chromosome may serve to mimic the effects of a new genomic environment for one of the paralogs, akin to that for paralogs residing on different chromosomes. 96 of 130 (~74%) duplicates pairs within this dataset comprised paralogs residing on the same chromosome. For this subset of duplicates pairs, I further investigated if (i) the extent of genomic distance between paralogs on the same chromosome (in bp), and (ii) transcriptional orientation of paralogs (two nominal categories; “same” if +/+ or −/−; “opposite” if +/− or −/+) has any discernible influence on their degree of sequence asymmetry at both the nucleotide and amino acid level. The average and median distances between paralogs on the same chromosome were 1,089,919 and 10,805 bp, respectively. 44 and 56% of duplicate pairs comprised paralogs in the same versus opposite transcriptional orientation, respectively.

Multiple regression analysis found no evidence that sequence asymmetry of paralogs is influenced by (i) genomic distance on the same chromosome, and (ii) the transcriptional orientation of paralogs. At the nucleotide sequence level, the whole model test was nonsignificant (*R*-square = 0.02734; *p*-value of whole model test = 0.2755) and neither parameter displayed a significant contribution to standardized nucleotide asymmetry among paralogs (*p*-value for genomic distance between paralogs on same chromosome = 0.6655; *p*-value for transcriptional orientation of paralogs on same chromosome = 0.1319). Likewise, at the amino acid sequence level, the whole model test was nonsignificant (*R*-square = 0.01235; *p*-value of whole model test = 0.346) and neither parameter displayed a significant contribution to standardized amino acid asymmetry among paralogs (*p*-value for genomic distance between paralogs on same chromosome = 0.9201; *p*-value for transcriptional orientation of paralogs on same chromosome = 0.1504).

### Duplication span of paralogs is correlated with structural category of paralogs

**Figure 5:**
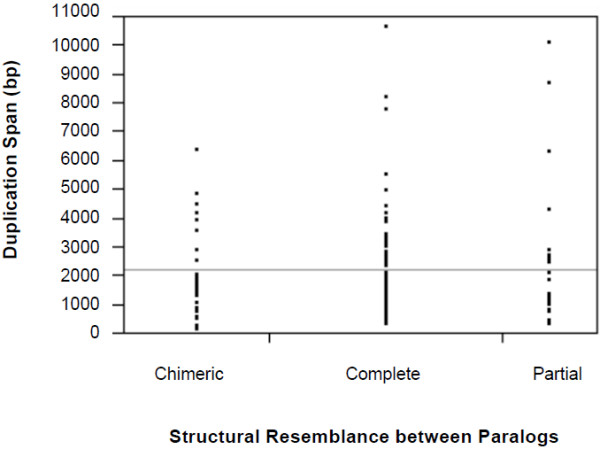
**Side-by-side vertical dot plots displaying the distribution of duplication spans for three structural categories of gene duplicates, namely *complete, partial *and *chimeric *duplicates (n = 64, 26, and 40 duplicate pairs, respectively).** The horizontal line across the graph shows the overall mean of all the observations.

In the preceding results, it was demonstrated that the duplication span (length of the duplicated sequence tract) had a significant influence on sequence asymmetry of *C. elegans* paralogs at both the nucleotide and amino acid sequence level. Duplication span values for duplicate pairs in this dataset ranged from 128 – 10,646 bp with a median value of 1,670 bp. Duplication events with attenuated spans are, on average, less likely to capture the entire coding sequence and/or regulatory sequences in the flanking regions of the ancestral copy [13,24,35,44] and this, in turn, may facilitate greater sequence asymmetry between paralogs. Indeed, this study has demonstrated a negative association between paralog asymmetry and duplication span. Although the structure of gene duplicates did not have a significant contribution to sequence asymmetry of paralogs based on the preceding multiple regression analysis, I further tested the degree of association between duplication span and the degree of structure resemblance between paralogs (*complete*, *partial* or *chimeric* duplicates). The duplication span of *complete* duplicates (*n* = 64 pairs) ranged from 330 – 10,646 bp with a median value of 1958 bp. The distribution of duplication spans for *partial* duplicates (*n* = 26 pairs) was similar to that of *complete* duplicates, with a range of 320 – 10,091 bp but with a lower median value of 1814 bp. Intriguingly, *chimeric* duplicates as a group (*n* = 40 pairs) possessed a greatly contracted range of duplications spans (128 – 6,335 bp) relative to *complete* and *partial* duplicates, with a median value of 1,347 bp (Figure 5). A Kruskal-Wallis test demonstrated that the three structural categories of gene duplicates differ significantly with respect to their distributions of duplication span (*H* = 6.3047; *p*-value = 0.0428) with structurally heterogeneous gene duplicates (*partial* and *chimeric*) possessing diminished duplication spans relative to homogeneous/*complete* duplicate pairs.

### Derived copies show accelerated sequence evolution at the amino acid level

Structural and syntenic comparisons of *C. elegans* paralogs to a single-copy ortholog in the *C. briggsae* genome enabled inference of the ancestral versus derived copy within 37 *C. elegans* duplicate pairs. In 30 of the 37 cases, the identity of the ancestral *C. elegans* paralog was established by the presence of greater exon-intron structure conservation with the *C. briggsae* ortholog [36]. In the case of 11 duplicates pairs with the focal paralogs residing on different chromosomes and an available outgroup sequence (single-copy ortholog in *C. briggsae* or another more evolutionary distant paralog in *C. elegans*), the *C. elegans* copy residing on the same chromosome as the outgroup was designated the ancestral copy. The dataset comprised an unbiased sampling of gene duplicate pairs with respect to both (i) category of structural resemblance (nine *complete*, 12 *partial*, and 16 *chimeric*) as well as (ii) chromosomal location of paralogs (24 and 23 pairs comprising paralogs on the same versus different chromosome(s), respectively). While the role of multiple translocation events cannot be discounted in the alteration of the ancestral locations of paralogs, it should be mentioned that with respect to four duplicate pairs for whom both structural and syntenic data were simultaneously available, both sources of data independently identified the same *C. elegans* paralog as the ancestral copy within the focal duplicate pair. Standardized asymmetry between the ancestral and derived copy across all 37 pairs was tested via a Wilcoxon-signed ranks test at both the nucleotide and amino acid sequence level. There was no significant difference in the rates of molecular evolution of the ancestral and derived copies at the nucleotide level (*T* = −80.5; *p-value* = 0.153). However, collectively, the derived copies were found to possess accelerated rates of molecular evolution at the amino acid level (*T* = −90.0; *p-value* = 0.021).

## Discussion

Although the role of gene duplication in the emergence of novel traits is firmly established, the genomic and/or evolutionary factors implicated in paralogs assuming divergent functional trajectories continue to evoke scrutiny. Ohno [14] envisioned gene duplication as spawning gene copies bearing sequence and functional redundancy to their ancestral counterparts. The gradual accumulation of mutations under conditions of relaxed selective constraints in one paralog enabled it to embark on a novel evolutionary trajectory leading to one of two alternative fates (neofunctionalization or nonfunctionalization). At the sequence level, both fates of neofunctionalization and nonfunctionalization are expected to leave a molecular signature of asymmetric rates of sequence divergence between the two paralogs [30]. Selection for greater gene dosage would lead to the retention of both paralogs with roughly symmetric rates of sequence divergence [24], unless selection for greater dosage is on the gene’s lesser auxiliary activities, in which case we might also expect asymmetric rates of sequence divergence [45]. Lastly, in addition to gene dosage, the process of subfunctionalization can also contribute to the retention of both paralogs in the genome [46], although it makes no predictions about the patterns of sequence evolution of the paralogs given its stochastic nature. Subfunctionalization could lead to a molecular signature of roughly equal rates of symmetrical evolution of paralogs if both copies equally divide ancestral subfunctions [30,47]. Conversely, unequal partitioning of ancestral subfunctions between paralogs would manifest as asymmetric rates of molecular sequence divergence.

Because the two paralogs were expected to be functionally identical under Ohno’s model [14], either copy could take on the role of maintaining the ancestral function, freeing the other redundant copy to embark on a novel evolutionary trajectory [1]. It is now evident that Ohno’s model of gene duplicate diversification has been rendered overly simplistic with respect to its predictions [1]. Most small-scale gene duplication events seldom yield duplicates that bear complete identity to the ancestral locus with respect to exon-intron structure, genomic neighborhood and the repertoire of regulatory elements [13,24,35,36,44]. This implies that a substantial fraction of gene duplicates may not meet the standard of functional equivalency at birth, an integral component of Ohno’s model for gene duplicate evolution and diversification [14].

This paper describes an analysis aimed to identify the major genomic correlates of sequence asymmetry among evolutionarily recent gene paralogs in the model eukaryote *Caenorhabditis elegans*. A handful of studies have tested the influence of some genomic correlates on sequence asymmetry between paralogs [30,31,34,47]. Interestingly, these studies focused on paralogs residing within mammalian genomes, which are known to have high origins of gene duplicates via retrotransposition [48-50]. As such, these studies explored the dependence of rate asymmetry on the mechanism of duplication (DNA-based *versus* RNA-based) and the extent of syntenic preservation given that duplication by retrotransposition entails massive changes in the genomic neighborhood of one paralog with respect to chromosomal location and a lack of proximity of ancestral regulatory sequences. The dataset analyzed here only contains two putative cases of RNA-based gene duplication (2/130 or 1.5%), namely duplicate pairs C54C6.1/W01D2.1 and B0035.2/C47A4.1 [36]. Hence, I focus on duplicates in a genome where DNA-mediated duplications far outnumber those formed via retrotransposition, providing a crucial contrast to preceding studies by investigating whether the evolutionary dynamics of DNA-based duplicates are influenced by similar genomic correlates as RNA-mediated duplications. Furthermore, this study represents a more comprehensive effort to explore the potential contribution of four predictor variables towards sequence asymmetry among paralogs.

I provide evidence that sequence asymmetry among paralogs (at both the nucleotide and amino acid levels) stems from three dominant determinants, namely (i) the evolutionary age of duplicates (K_*S*_), (ii) the chromosomal location of paralogs, and (iii) the duplication span. Overall, these three predictors accounted for ~19.4% of the variation in rate asymmetry at the nucleotide level among *C. elegans* paralogs. In concordance with the nucleotide sequence results, the very same three predictors appear to significantly influence asymmetry between paralogs at the amino acid level, accounting for ~13% of the variation. The implications of each of these three predictors for sequence and functional diversification of paralogs are further elaborated in the following sections.

The results demonstrate that ~13.1% (17 of 130 pairs) and 7% (9 of 130 pairs) of *C. elegans* gene duplicate pairs with K_*S*_ values ranging from 0 to 0.13 displayed significant rate asymmetry at the nucleotide and amino acid level based on Tajima’s relative rate test, respectively. The majority of duplicate pairs with significant nucleotide sequence asymmetry had K_*S*_ values of ~0.03 or greater. This is not to say that mutations accumulated in the early evolutionary life of duplicates have no discernible influence on dictating rate asymmetry between paralogs. On the contrary, it is very likely that the evolutionary trajectories of paralogs are altered at birth or in evolutionary infancy given the possibility that the earliest mutations have a disproportionately larger effect in dictating divergent evolutionary trajectories for paralogs. Given that the duplicates in this study are thought to be of recent evolutionary origin based on their low K_*S*_ values, this result demonstrates that paralogs start exhibiting the molecular signature of asymmetric evolution remarkably early in their evolutionary existence. Although gene conversion cannot be ruled out as a contributing factor to low synonymous divergence between paralogs and may serve to homogenize at least a fraction of the duplicate pairs within this dataset [51,52], it can at best only delay the inevitable sequence asymmetry in the molecular evolution of paralogs. Over longer evolutionary periods, this evident signature of divergent molecular evolution in the early life of paralogs may come to be obscured by later mutational events [24,47].

This study provides evidence that the movement of one paralog to a different chromosome contributes significantly to sequence asymmetry among *C. elegans* paralogs. This result is similar to the conclusion of the Cusack and Wolfe study [30], providing independent confirmation of the influence of altered genomic environment on paralog sequence asymmetry in a population of gene duplicates primarily created by DNA-mediated duplication events. The relocation of one paralog to a novel genomic environment engenders multifold effects. For one, paralogs residing in genomically remote locations relative to the cognate copy are likely more stable due to a decreased probability of loss due to unequal exchange [35]. Second, the genomic proximity of paralogous genes is thought to facilitate gene conversion [51,53-55]. Hence, distant paralogs are more likely to embark on a novel evolutionary trajectory given that the probability of homogenization with the ancestral copy due to gene conversion events is reduced [47]. Third, paralogs residing in close genomic proximity are likely to share their ancestral regulatory elements [24,35] the ancestral gene neighborhood (local synteny) as well as short-range chromatin effects [30,56], which are thought to cumulatively serve to increase the likelihood of symmetrical rates of paralog evolution under both selective [57-59] or neutral regimes [60]. Conversely, the relocation of one paralog to a novel genomic locale has the potential to initiate a cascade of alterations to its ancestral regulatory repertoire and genic neighborhood, thereby increasing its probability of embarking on a divergent evolutionary trajectory [24]. Indeed, faster rates of molecular evolution have additionally been detected in relocated paralogs in bacteria [61], paralogs originating from small-scale duplication events [23] and those residing in a low-recombination environment in yeast [56], and in vertebrates [30,34,47,62].

The six chromosomes comprising the *C. elegans* genome range in size from 13.78 – 20.92 Mbp with an average length of 16.71 Mbp. Although the chromosomes are relatively small in size, there is a possibility that paralogs located at either end of a chromosome may have patterns of sequence asymmetry akin to paralogs residing on different chromosomes, with a possible increase in rate asymmetry between paralogs the further apart they are located on the same chromosome. Additionally, chromosomal rearrangements such as inversions change the transcriptional orientation of paralogs with the added potential to alter their exon-intron structure as well as the spatial organization of their *cis*-regulatory elements. A large fraction of evolutionarily young gene duplicate pairs within the *C. elegans* genomes comprise paralogs residing on the same chromosome [35], comprising approximately 74% (96 of 130) of the gene duplicate pairs within this study. This subset of duplicate pairs was further analyzed to determine if transcriptional orientation of paralogs and the genomic distance (bp) between the paralogs influences rate asymmetry of paralogs at the nucleotide and amino acid level. I found no evidence for increased sequence asymmetry at the nucleotide or amino acid level with increasing genomic distance between paralogs on the same chromosome. In this regard, the results of this study conflict with that of Cusack and Wolfe [30] which demonstrated that rodent paralogs >5 gene loci apart on the same chromosome displayed rate asymmetry at levels similar to paralogs residing on different chromosomes. Nor did the transcriptional orientation of paralogs on the same chromosome contribute to rate symmetry at the sequence level; that is, duplicate pairs comprising paralogs in opposite transcriptional orientation on the same chromosome did not exhibit greater rate asymmetry relative to their counterparts with the same transcriptional orientation.

A third predictor, the duplication span (length of the duplicated sequence tract), exerts a significant influence on rate asymmetry between paralogs at both the nucleotide and amino acid level. The shorter the duplicate span, the greater the sequence rate asymmetry between paralogs. In a previous study of *C. elegans* duplicates, it was demonstrated that the frequency distribution of duplication spans arising from small-scale duplication events followed an L-shaped distribution with a preponderance of short duplicated tracts that likely failed to encompass entire genes [35]. Attenuated duplication spans likely have at least two important consequences for the future evolution of newborn gene duplicates: (i) a failure to encompass the entire ancestral coding sequence results in the creation of a structurally heterogeneous daughter copy (*partial* or *chimeric* duplicates), and (ii) flanking regions containing ancestral *cis*-regulatory elements may not be inherited by the daughter copy, thereby priming the daughter copy for a divergent evolutionary trajectory at the very onset [24]. Indeed, the negative correlation between duplication span and sequence asymmetry in this study provides indirect evidence for the hypothesis that attenuated duplication spans create daughter copies that violate the assumption of sequence and functional redundancy with the ancestral locus, thereby initiating novel evolutionary trajectories for the derived loci. It remains to be determined what fraction of the time these accelerated rates of sequence evolution in the derived copy ultimately result in nonfunctionalization, subfunctionalization or neofunctionalization.

~15% (20 of 130) gene duplicate pairs in this study comprised one paralog designated a pseudogene. Duplicate pairs wherein one copy or both copies have unique exonic sequence to the exclusion of the other copy (*partial* and *chimeric* duplicates, respectively) may be expected to display higher rates of pseudogenization, given the massive alterations to the ancestral exon-intron structure entailed during such incomplete duplication events. Contrary to expectations, there was no significant difference in the frequency of pseudogene containing duplicate pairs among the three structural categories of duplicates (*complete, partial* and *chimeric*), nor were pseudogene-containing duplicate pairs more likely to display asymmetric rates of sequence evolution relative to pairs comprising both putatively functional paralogs. While pseudogenes have traditionally been considered nonfunctional sequences of genomic DNA that are prone to the accumulation of degenerative mutations, detailed empirical analyses of pseudogene evolution have revealed numerous instances of extremely conserved, transcriptionally active and functional pseudogenes that may contribute to the generation of genetic diversity [63-66]. Although pseudogene-containing duplicate pairs represent a small sample within this dataset and likely suffer from issues of statistical power, this study could not reject the null hypothesis that patterns of sequence evolution among duplicate pairs containing a pseudogene and those with functional paralogs are similar.

Lastly, this study explores the intriguing question as to which paralog, ancestral or derived, takes on a divergent evolutionary trajectory. Under Ohno’s model, since paralogs were considered to be functionally redundant at birth, either copy is equally likely to embark on a novel evolutionary trajectory while its sister copy maintains the ancestral function [1]. This hypothesis was tested in a subset of 37 *C. elegans* duplicate pairs wherein the ancestral *versus* the derived copy was identified based on structural and syntenic comparisons with a single-copy ortholog in the genome of a congener species, *C. briggsae*. While there were no detectable differences in the rates of molecular evolution between paralogs at the nucleotide level, the derived copies collectively exhibited accelerated rates of evolution at the amino acid level. Similar results of accelerated sequence evolution of derived copies relative to their ancestral counterparts have been demonstrated for paralogs originating from small-scale duplication events in *Saccharomyces cerevisiae*[23] and in duplicates from the neo-X chromosome in *Drosophila pseudoobscura*[67]. This study and others provide accumulating evidence that the majority of gene duplicates violate Ohno’s assumption of functional equivalency at birth and the derived copy is primed for a divergent evolutionary trajectory by changes in its structure and genomic context at inception. Even duplicates with complete homology over their coding regions are likely distinguishable based on differences in their regulatory regions. This also suggests that natural selection has the patrolling ability to differentiate between the ancestral and derived locus and potentially purge deleterious mutations that accumulate in the ancestral paralog in charge of maintaining the ancestral function.

## Conclusion

In conclusion, this genome-wide study of evolutionarily young duplicates stemming primarily from DNA-mediated small-scale duplication events demonstrates that genomic relocation to a new chromosome has important consequences for asymmetric divergence of paralogs, akin to paralogs arising from RNA-mediated duplication events. Another genomic determinant, the duplication span, is identified as exerting a significant negative effect on sequence rate asymmetry among paralogs. The shorter the duplication span, the greater the probability of altering the local genomic context of the duplicated copy owing to a failure to encapsulate the entire genic neighborhood of the ancestral locus. Attenuated duplication spans, in addition to replicating only a partial tract of the open reading frame, may also fail to encompass ancestral *cis*-regulatory regions. In turn, the new genomic environment attained by the duplicate copy may accelerate the path to nonfunctionalization if it fails to acquire a new promoter and/or complete its truncated reading frame by recruiting new neighborhood sequence. Alternatively, the duplicate copy may embark on a novel evolutionary trajectory towards neofunctionalization by gaining novel promoters and/or coding sequence tracts. Sequence rate asymmetry between paralogs is correlated with asymmetric functional divergence [22,68]. For example, yeast paralogs with accelerated evolutionary rates at the sequence level tend to have greater dispensability, fewer protein-protein interactions [22], reduced selection for codon usage and lowered mRNA abundance [23]. The relationship between accelerated rates of molecular evolution in paralogs and their evolutionary fate remains to be determined. Are a disproportionately larger fraction of such gene copies with molecular signatures of accelerated sequence evolution likely to endure a fate of extinction/pseudogenization? Or are they equally likely to evolve into neofunctionalized genes?

## Competing interest

The author declares that she has no competing interests.

## Author’s contribution

VK conceived and designed the experiment, collected the data, performed the data analysis and wrote the manuscript. The author has approved the final manuscript.

## Supplementary Material

Additional file 1: Table S1 Tajima’s relative rate test results for nucleotide sequences of 130 C. elegans gene duplicate pairs using a single-copy ortholog in a related genome as outgroup. The Outgroup column lists the genomic source of the single-copy ortholog used as an outgroup sequence in the Tajima’s relative rate test: *Cbren = C. brenneri, Cbrig = C. briggsae, Celeg = C. elegans, Cjapo = C. japonica, Crema = C. remanei.* Click here for file

Additional file 2: Table S2 Tajima’s relative rate test results for amino acid sequences of 130 C. elegans gene duplicate pairs using a single-copy ortholog in a related genome as outgroup. The Outgroup column lists the genomic source of the single-copy ortholog used as an outgroup sequence in the Tajima’s relative rate test*: Cbren = C. brenneri, Cbrig = C. briggsae, Celeg = C. elegans, Cjapo = C. japonica, Crema = C. remanei.* Click here for file
