# Supplementary material for: To the beat of a different drum: determinants implicated in the asymmetric sequence divergence of Caenorhabditis elegans paralogs
Source: BMC Evol Biol. 2013 Mar 27;13:73. doi: 10.1186/1471-2148-13-73 (PMC3637608; doi:10.1186/1471-2148-13-73)
Supplement: Additional file 2: Table S2 — Tajima’s relative rate test results for amino acid sequences of 130 C. elegans gene duplicate pairs using a single-copy ortholog in a related genome as outgroup. The Outgroup column lists the genomic source of the single-copy ortholog used as an outgroup sequence in the Tajima’s relative rate test: Cbren = C. brenneri, Cbrig = C. briggsae, Celeg = C. elegans, Cjapo = C. japonica, Crema = C. remanei. [file 1471-2148-13-73-S2.doc]

## Additional file 2: Table S2. Tajima’s relative rate test results for amino acid sequences of 130 C. elegans gene duplicate pairs using a single-copy ortholog in a related genome as outgroup. The Outgroup column lists the genomic source of the single-copy ortholog used as an outgroup sequence in the Tajima’s relative rate test: Cbren = C. brenneri, Cbrig = C. briggsae, Celeg = C. elegans, Cjapo = C. japonica, Crema = C. remanei.

|  | ***C. elegans***  ***Paralog A*** | ***C. elegans***  ***Paralog B*** | ***Outgroup*** | ***χ^2^*** | ***p-value*** | ***Unique Amino Acid Sites*** | | ***Asymmetry***  ***|(A-B)|*** | ***Asymmetry/Site*** |
| --- | --- | --- | --- | --- | --- | --- | --- | --- | --- |
|  |  |  |  |  |  | ***A*** | ***B*** |  |  |
| 1 | C48E7.7 | T08B2.12 | Crema_CRE29426 | 1.00 | 0.317 | 1 | 0 | 1 | 0.0031 |
| 2 | C54C6.1 | W01D2.1 | Cbrig_CBG04239 | 1.00 | 0.317 | 1 | 0 | 1 | 0.0045 |
| 3 | F10E7.11 | T07F8.4 | Cbrig_CBG13072 | 1.00 | 0.317 | 0 | 1 | 1 | 0.0022 |
| 4 | H16D19.3 | T07D10.3 | Crema_CRE14172 | 0.00 | 1.000 | 0 | 0 | 0 | 0.0025 |
| 5 | Y24F12A.3 | Y24F12A.4 9 (pseudo) | Celeg_H37A05.4 | 1.00 | 0.317 | 1 | 0 | 1 | 0.0039 |
| 6 | D2045.2 | H04D03.3 | Cbren_CBN22673 | 1.00 | 0.317 | 0 | 1 | 1 | 0.0010 |
| 7 | C24A8.4 | F14H12.4b | Cbrig_CBG14395 | 1.00 | 0.317 | 1 | 0 | 1 | 0.0012 |
| 8 | C45G9.4 | C45G9.9 | Cbrig_CBG23024 | 0.00 | 1.000 | 1 | 1 | 0 | 0.0062 |
| 9 | F38A5.5 | F38A5.12 | Celeg_H04M03.2 | 1.00 | 0.317 | 1 | 0 | 1 | 0.0050 |
| 10 | F58D12.1 | K08D10.11 | Celeg_F40E3.3 | 1.00 | 0.317 | 0 | 1 | 1 | 0.0069 |
| 11 | ZC412.6 | ZC412.7 | Crema_CRE01691 | 0.00 | 1.000 | 0 | 0 | 0 | 0.0127 |
| 12 | C04C3.4 | T07F10.5 | Celeg_W05G11.2 | 2.00 | 0.157 | 0 | 2 | 2 | 0.0231 |
| 13 | F08F1.9 | F59H6.7 | Cbrig_CBG06936 | 0.33 | 0.564 | 2 | 1 | 1 | 0.0191 |
| 14 | K09H9.1 | Y54E10A.12 | Crema_CRE30104 | 3.00 | 0.083 | 3 | 0 | 3 | 0.0280 |
| 15 | C27C7.2 (pseudo) | F41D3.8 | Crema_CRE18586 | 0.00 | 1.000 | 0 | 0 | 0 | 0.0484 |
| 16 | F12A10.2 | F12A10.6 | Celeg_ZK177.2 | 3.00 | 0.083 | 3 | 0 | 3 | 0.0535 |
| 17 | F41D3.8 | ZK1025.4 | Crema_CRE18579 | 1.00 | 0.317 | 0 | 1 | 1 | 0.0484 |
| 18 | C18A11.6 | Y39B6A.50 | Celeg_F11A5.6 | 0.00 | 1.000 | 2 | 2 | 0 | 0.0622 |
| 19 | F19G12.2 | F58F9.1 | Cbren_CN37089 | 3.00 | 0.083 | 0 | 3 | 3 | 0.0055 |
| 20 | ZK1127.6 | ZK1127.9A | Cbrig_CBG11210 | 0.00 | 1.000 | 0 | 0 | 0 | 0.0023 |
| 21 | F31A3.2 | F31A3.4 | Cjapo_CJA11253 | 0.00 | 1.000 | 0 | 0 | 0 | 0.0035 |
| 22 | H05C05.3 | Y54G2A.20 | Celeg_F52C6.14 | 1.00 | 0.317 | 3 | 1 | 2 | 0.0095 |
| 23 | F59E12.4b | F59E12.5b | Cbrig_CBG06960 | 0.00 | 1.000 | 2 | 2 | 0 | 0.0040 |
| 24 | 3R5.2 (pseudo) | K08E3.8 | Cbrig_CBG18261 | 3.00 | 0.083 | 3 | 0 | 3 | 0.0133 |
| 25 | C36A4.5 | F42A6.3 (pseudo) | Celeg_F32A7.5a | 0.04 | 0.847 | 13 | 14 | 1 | 0.0276 |
| 26 | R102.1 | Y41E3.6 | Cbrig_CBG04448 | 0.00 | 1.000 | 0 | 0 | 0 | 0.0040 |
| 27 | Y73F8A.12 | Y73F8A.14 | Celeg_C34F11.2 | 0.00 | 1.000 | 2 | 2 | 0 | 0.0131 |
| 28 | Y51H4A.17a | Y51H4A.18 (pseudo) | Cbrig_CBG13675 | 0.00 | 1.000 | 0 | 0 | 0 | 0.0043 |
| 29 | Y51H4A.17a | Y51H4A.20 (pseudo) | Cbrig_CBG13675 | 1.00 | 0.317 | 0 | 1 | 1 | 0.0040 |
| 30 | Y51H4A.17 | Y51H4A.19 (pseudo) | Cbrig_CBG13675 | 1.00 | 0.317 | 0 | 1 | 1 | 0.0068 |
| 31 | F20D1.4 | F45E4.2 | Crema_CRE12527 | 0.33 | 0.564 | 2 | 1 | 1 | 0.0152 |
| 32 | T02G5.11 | ZK1127.1 | Cbren_CBN32839 | 0.00 | 1.000 | 1 | 1 | 0 | 0.0050 |
| 33 | T24A6.1 | ZK678.3 | Cbrig_CBG06936 | 1.00 | 0.317 | 0 | 1 | 1 | 0.0085 |
| 34 | C43E11.5 | C50F2.5 | Cbrig_CBG08352 | 0.33 | 0.564 | 1 | 2 | 1 | 0.0049 |
| 35 | C55A1.4 | C55A1.7 | Cbrig_CBG04677 | 0.00 | 1.000 | 0 | 0 | 0 | 0.0000 |
| 36 | C14C11.1 | ZC317.6 | Cbrig_CBG09348 | 0.00 | 1.000 | 1 | 1 | 0 | 0.0052 |
| 37 | B0546.5 | F35E2.1 | Cbrig_CBG04040 | 2.67 | 0.102 | 1 | 5 | 4 | 0.0119 |
| 38 | C49C3.1 | Y43D4A.1 | Cbrig_CBG00451 | 0.00 | 1.000 | 0 | 0 | 0 | 0.0000 |
| 39 | F20B6.6 | Y71F9AL.4 | Crema_CRE25785 | 1.00 | 0.317 | 0 | 1 | 1 | 0.0099 |
| 40 | C36C9.4 | T25D1.2 | Cbren_CBN06255 | 2.00 | 0.157 | 2 | 0 | 2 | 0.0083 |
| 41 | C27F2.2 | F17C8.6 | Cbren_CBN15067 | 1.00 | 0.317 | 0 | 1 | 1 | 0.0015 |
| 42 | F35C11.2 | M05D6.3 | Crema_CRE01001 | 2.00 | 0.157 | 0 | 2 | 2 | 0.0047 |
| 43 | K01D12.15 | K01D12.7 | Cbrig_CBG13849 | 0.00 | 1.000 | 0 | 0 | 0 | 0.0000 |
| 44 | VH15N14R.1 | Y24F12A.3 | Celeg_Y51H7C.15 | 0.00 | 1.000 | 0 | 0 | 0 | 0.0188 |
| 45 | F33H1.2 | T09F3.3 | Celeg_K10B3.8 | 0.00 | 1.000 | 1 | 1 | 0 | 0.0030 |
| 46 | F44E7.2 | K09H11.7 | Celeg_C45E5.1 | 0.00 | 1.000 | 0 | 0 | 0 | 0.0014 |
| 47 | C02B8.2 | F31A9.4 | Crema_CRE07574 | 0.00 | 1.000 | 0 | 0 | 0 | 0.0172 |
| 48 | B0273.3 | F18A11.6 (pseudo) | Cbren_CBN17858 | 0.33 | 0.564 | 2 | 1 | 1 | 0.0093 |
| 49 | B0546.5 | F16D3.6 | Cbrig_CBG04040 | 1.00 | 0.317 | 1 | 0 | 1 | 0.0058 |
| 50 | C17C3.7 | C17C3.10 | Celeg_C17C3.8 | 0.00 | 1.000 | 0 | 0 | 0 | 0.0061 |
| 51 | T07F10.5 | ZK678.3 | Celeg_F59H6.7 | 1.00 | 0.317 | 1 | 0 | 1 | 0.0302 |
| 52 | H31G24.3 | Y43E12A.3 | Crema_CRE07121 | 3.00 | 0.083 | 3 | 0 | 3 | 0.0155 |
| 53 | C03B8.2 | C04C3.4 | Celeg_T07F10.5 | 2.67 | 0.102 | 5 | 1 | 4 | 0.0362 |
| 54 | H31G24.4 | Y43E12A.1 | Cbrig_CBG17647 | 0.50 | 0.480 | 3 | 5 | 2 | 0.0233 |
| 55 | ZK637.12 | ZK637.15 | Crema_CRE16397 | 2.00 | 0.157 | 0 | 2 | 2 | 0.0070 |
| 56 | C27A7.6 | Y43D4A.4a | Cbrig_CBG23388 | 1.00 | 0.317 | 0 | 1 | 1 | 0.0016 |
| 57 | F16D3.6 | F35E2.1 | Crema_CRE03763 | 7.00 | 0.008 | 0 | 7 | 7 | 0.0116 |
| 58 | C10G11.9 | T27A3.4 | Cbren_CBN05113 | 1.00 | 0.317 | 0 | 1 | 1 | 0.0051 |
| 59 | C36A4.5 | F25D7.4 | Celeg_F32A7.5 | 5.83 | 0.016 | 21 | 8 | 13 | 0.0180 |
| 60 | C37C3.3 | Y61A9LA.5 (pseudo) | Cbrig_CBG04319 | 2.00 | 0.157 | 6 | 12 | 6 | 0.0617 |
| 61 | M04F3.3 | W09C3.1 | Cbrig_CBG14903 | 2.00 | 0.157 | 0 | 2 | 2 | 0.0052 |
| 62 | C03B8.2 | W05G11.2 | Crema_CRE05114 | 3.00 | 0.083 | 0 | 3 | 3 | 0.0685 |
| 63 | B0379.2 | Y106G6E.3 (pseudo) | Cbrig_CBG03810 | 0.33 | 0.564 | 1 | 2 | 1 | 0.0101 |
| 64 | F25D7.4 | F42A6.3 (pseudo) | Crema_CRE20747 | 4.45 | 0.035 | 2 | 9 | 7 | 0.0197 |
| 65 | F32B5.1 | W10C8.5 | Cbrig_CBG04232 | 3.00 | 0.083 | 3 | 0 | 3 | 0.0032 |
| 66 | C14C11.2 | ZC317.7 | Cbrig_CBG09349 | 1.80 | 0.180 | 4 | 1 | 3 | 0.0054 |
| 67 | F54H12.4 | Y57G11C.18 | Crema_CRE21823 | 0.00 | 1.000 | 1 | 1 | 0 | 0.0192 |
| 68 | F34H10.1 | K08C9.7 | Cbren_CBN24339 | 2.00 | 0.157 | 0 | 2 | 2 | 0.0261 |
| 69 | ZK899.5 | ZK899.6 | Crema_CRE22335 | 1.00 | 0.317 | 1 | 0 | 1 | 0.0194 |
| 70 | C38C10.4 | F22B7.13 | Cbrig_CBG10100 | 0.69 | 0.405 | 8 | 5 | 3 | 0.0154 |
| 71 | D1022.3 | K02A2.1 | Crema_CRE26129 | 0.00 | 1.000 | 1 | 1 | 0 | 0.0192 |
| 72 | F57F4.3 | F57F4.4 | Cbrig_CBG18927 | 0.60 | 0.439 | 6 | 9 | 3 | 0.0063 |
| 73 | C14C11.6 | ZC317.1 | Cbrig_CBG09254 | 2.00 | 0.157 | 0 | 2 | 2 | 0.0063 |
| 74 | F41G4.5 | ZC15.1 | Cbren_CBN04572 | 2.00 | 0.157 | 2 | 0 | 2 | 0.0137 |
| 75 | C01G12.2 | C01G12.6 | Celeg_C01G12.1 | 1.00 | 0.317 | 0 | 1 | 1 | 0.0061 |
| 76 | F15E6.1 | Y51H4A.12 | Crema_CRE31573 | 0.29 | 0.593 | 8 | 6 | 2 | 0.0128 |
| 77 | C08E3.1 | C08E.13 | Celeg_Y105C5A.12 | 0.00 | 1.000 | 0 | 0 | 0 | 0.0000 |
| 78 | F52C6.2 | F52C6.3 | Celeg_F52C6.4 | 0.67 | 0.414 | 4 | 2 | 2 | 0.0547 |
| 79 | F32B6.5 | T13F2.9 (pseudo) | Cbrig_CBG0225 | 0.00 | 1.000 | 3 | 3 | 0 | 0.0308 |
| 80 | W03D8.9 | W03D8.10 | Cbrig_CBG09313 | 1.00 | 0.317 | 3 | 1 | 2 | 0.0096 |
| 81 | C29F7.4 | C29F7.5 | Celeg_F26A1.2 | 0.00 | 1.000 | 2 | 2 | 0 | 0.0119 |
| 82 | F13B6.2 | F46F5.8 | Cbrig_CBG13505 | 0.33 | 0.564 | 1 | 2 | 1 | 0.0829 |
| 83 | C03A7.7 | C03A7.4 | Celeg - C03A7.8 | 0.20 | 0.655 | 3 | 2 | 1 | 0.0063 |
| 84 | Y48A6C.1 (pseudo) | Y48A6C.4 | Cbrig_CBG13183 | 1.00 | 0.317 | 1 | 0 | 1 | 0.0023 |
| 85 | M01G12.2 | M01G12.5 | Crema_CRE28616 | 0.00 | 1.000 | 0 | 0 | 0 | 0.0020 |
| 86 | H12D21.1 | W06A7.5 | Cbren_CBN19867 | 1.00 | 0.317 | 1 | 0 | 1 | 0.0241 |
| 87 | W06A7.5 | ZC412.7 | Cbren_CBN19867 | 1.00 | 0.317 | 0 | 1 | 1 | 0.0241 |
| 88 | C25F9.4 | M04C3.2 | Celeg_Y43F8B.14 | 1.38 | 0.239 | 16 | 10 | 6 | 0.0324 |
| 89 | C49A9.1 | E02H9.4 | Cbrig_CBG05496 | 0.00 | 1.000 | 0 | 0 | 0 | 0.0224 |
| 90 | W06A7.5 | ZC412.6 | Cbren_CBN19867 | 2.00 | 0.157 | 0 | 2 | 2 | 0.0378 |
| 91 | W09D6.4 | Y47D3A.13 | Cbrig_CBG13192 | 1.00 | 0.317 | 1 | 3 | 2 | 0.0171 |
| 92 | C33E10.10 | T25G12.2 | Cbrig_CBG15942 | 0.00 | 1.000 | 1 | 1 | 0 | 0.0161 |
| 93 | C32H11.6 | C32H11.8 | Cbrig_CBG21288 | 0.50 | 0.480 | 3 | 5 | 2 | 0.0613 |
| 94 | T02G5.6 | ZK1127.11 | Cbrig_CBG24743 | 3.00 | 0.083 | 3 | 0 | 3 | 0.0129 |
| 95 | T06E4.8 | T06E4.9 | Celeg_T06E4.10 | 0.33 | 0.564 | 2 | 1 | 1 | 0.0375 |
| 96 | C32H11.6 | C32H11.11 | Cbrig_CBG21288 | 0.50 | 0.480 | 3 | 5 | 2 | 0.0569 |
| 97 | C27C7.1 | T16G12.3 | Celeg_H11L12.1 | 3.60 | 0.058 | 8 | 2 | 6 | 0.0298 |
| 98 | B0250.1 | B0250.7 (pseudo) | Cbrig_CBG05588 | 2.00 | 0.157 | 0 | 2 | 2 | 0.0224 |
| 99 | H11L12.1 | T16G12.3 | Celeg_Y54G2A.19 | 0.00 | 1.000 | 2 | 2 | 0 | 0.0308 |
| 100 | C47C12.4 | K08F11.5 | Cbrig_CBG01740 | 3.67 | 0.102 | 5 | 1 | 4 | 0.0112 |
| 101 | F53B2.2 | Y45F10B.1 | Cbrig_CBG02040 | 0.20 | 0.655 | 3 | 2 | 1 | 0.0235 |
| 102 | T28H11.1 | ZC477.7 | Cbrig_CBG20412 | 0.00 | 1.000 | 0 | 0 | 0 | 0.0022 |
| 103 | C54E10.1 | T08G5.9 | Cbrig_CBG02279 | 1.00 | 0.317 | 3 | 1 | 2 | 0.0374 |
| 104 | C37A5.2 | C37A5.4 | Celeg - C37A5.8 | 0.00 | 1.000 | 0 | 0 | 0 | 0.0188 |
| 105 | H06O01.4 (pseudo) | M05B5.1 | Celeg_K07F5.6 | 3.00 | 0.083 | 3 | 0 | 3 | 0.0150 |
| 106 | C33F10.1 | C33F10.11 | Cbrig_CBG09925 | 0.67 | 0.414 | 4 | 2 | 2 | 0.0478 |
| 107 | F38A5.5 | F38A5.9 | Cbrig_CBG05403 | 1.00 | 0.317 | 1 | 0 | 1 | 0.0104 |
| 108 | C06E7.1A | Y105C5B.12 (pseudo) | Celeg - C06E7.3a | 4.00 | 0.046 | 0 | 4 | 4 | 0.0046 |
| 109 | F38A5.9 | F38A5.12 | Cbrig_CBG05403 | 0.00 | 1.000 | 0 | 0 | 0 | 0.0052 |
| 110 | C28A5.6 | K09C6.2 | Cbrig_CBG18064 | 0.07 | 0.080 | 7 | 8 | 1 | 0.0415 |
| 111 | C03A7.7 | C03A7.8 | Celeg_R09F10.2 | 1.00 | 0.317 | 3 | 1 | 2 | 0.0107 |
| 112 | C25F9.6 | C25F9.7 | Celeg_C25F9.12 | 2.78 | 0.096 | 7 | 2 | 5 | 0.2016 |
| 113 | F31F6.1 | F31F6.3 | Celeg - F31F6.2 | 20.55 | 0.000 | 43 | 10 | 33 | 0.1329 |
| 114 | T19D12.1 | T19D12.2 | Cbrig_CBG11242 | 1.60 | 0.206 | 3 | 7 | 4 | 0.0244 |
| 115 | C03A7.2 | ZK262.8 | Celeg_F07B7.2 | 0.20 | 0.655 | 3 | 2 | 1 | 0.0193 |
| 116 | T28F3.8 | Y51H4A.24 (pseudo) | Cbrig_CBG00374 | 2.78 | 0.096 | 2 | 7 | 5 | 0.0199 |
| 117 | F42G8.8 | Y37A1B.5 | Cbrig_CBG18761 | 0.20 | 0.655 | 3 | 2 | 1 | 0.0235 |
| 118 | F07G6.3 | Y20C6A.3 (pseudo) | Celeg_Y67D8C.2 | 0.17 | 0.683 | 11 | 13 | 2 | 0.0746 |
| 119 | F55A3.3 | F55A3.7 | Cbrig_CBG12204 | 9.31 | 0.002 | 1 | 12 | 11 | 0.0154 |
| 120 | K09C6.7 | K09C6.8 | Celeg_T10B5.2 | 0.14 | 0.705 | 4 | 3 | 1 | 0.0131 |
| 121 | B0035.2 | C47A4.1 | Cbrig_CBG06069 | 0.00 | 1.000 | 0 | 0 | 0 | 0.0025 |
| 122 | F42A6.1 | Y55H10A.2 | Celeg__K03H6.4 | 1.00 | 0.317 | 0 | 1 | 1 | 0.0229 |
| 123 | F38A5.9 | F38A5.10 | Celeg - F38A5.12 | 1.00 | 0.317 | 0 | 1 | 1 | 0.0052 |
| 124 | C29A12.2 (pseudo) | F29G9.7 | Cbrig_CBG10639 | 0.60 | 0.439 | 9 | 6 | 3 | 0.0672 |
| 125 | T07F10.5 | W05G11.2 | Celeg_ZK678.3 | 0.14 | 0.705 | 3 | 4 | 1 | 0.0925 |
| 126 | F42F12.1 | F42F12.6 | Celeg_D1025.4 | 1.00 | 0.317 | 0 | 1 | 1 | 0.0043 |
| 127 | Y73F8A.22 | Y116A8C.1 | Celeg_Y73F8A.23 | 12.25 | 0.000 | 1 | 15 | 14 | 0.0927 |
| 128 | C06C3.4 | C06C3.9 | Crema_CRE01911 | 2.00 | 0.157 | 0 | 2 | 2 | 0.0067 |
| 129 | C56C10.3 | Y61A9LA.5 (pseudo) | Crema_CRE26519 | 35.37 | 0.000 | 2 | 41 | 39 | 0.1407 |
| 130 | C03A7.7 | C03A7.14 | Celeg_R09F10.2 | 9.00 | 0.003 | 9 | 0 | 9 | 0.0214 |
